# Phylogenetic and Physiological Diversity of Cultivable Actinomycetes Isolated From Alpine Habitats on the Qinghai-Tibetan Plateau

**DOI:** 10.3389/fmicb.2020.555351

**Published:** 2020-10-02

**Authors:** Aiai Ma, Xinfang Zhang, Kan Jiang, Changming Zhao, Junlin Liu, Mengdan Wu, Ying Wang, Mingming Wang, Jinhui Li, Shijian Xu

**Affiliations:** ^1^School of Life Sciences, Lanzhou University, Lanzhou, China; ^2^Life Science and Engineering College of Northwest University for Nationalities, Lanzhou, China; ^3^College of Agronomy, Gansu Agricultural University, Lanzhou, China

**Keywords:** actinomycetes, phylogenetic diversity, physiological activities, alpine habitat, Qinghai-Tibetan Plateau

## Abstract

Actinomycetes in extreme alpine habitat have attracted much attention due to their unique physiological activities and functions. However, little is known about their ecological distribution and diversity. Here, we explored the phylogenetic relationship and physiological heterogeneity of cultivable actinomycetes from near-root soils of different plant communities in the Laohu Ditch (2200 – 4200 m) and Gaize County area (5018 – 5130 m) on the Qinghai-Tibetan Plateau. A total of 128 actinomycete isolates were obtained, 16S rDNA-sequenced and examined for antimicrobial activities and organic acid, H_2_S, diffusible pigments, various extracellular enzymes production. Seventy three isolates of the total seventy eight isolates from the Laohu Ditch, frequently isolated from 2200 to 4200 m, were closely related to *Streptomyces* spp. according to the 16S rDNA sequencing, while four isolates within the genus *Nocardia* spp. were found at 2200, 2800, and 3800 m. In addition, one potential novel isolate with 92% sequence similarity to its nearest match *Micromonospora saelicesensis* from the GenBank database, was obtained at 2200 m. From the Gaize County area, fifty *Streptomyces* isolates varied in diversity at different sites from 5018 to 5130 m. The investigation of phenotypic properties of 128 isolates showed that 94.5, 78.9, 68, 64.8, 53, 51.6, 50, 36.7, 31.2, and 22.7% of the total isolates produced catalase, lipase 2, urease, protease, H_2_S, lipase 3, amylase, lipase 1, diffusible pigment and organic acid, respectively. The antimicrobial assays of the total isolates revealed that 5, 28, 19, and 2 isolates from *Streptomyces* spp. exhibited antimicrobial activity against *Escherichia coli*, *Staphylococcus aureus*, *Candida albicans*, and *Pseudomonas aeruginosa*, respectively. This study intends to bring helpful insights in the exploitation and utilization of alpine actinomycetes for novel bioactive compounds discovery.

## Introduction

Actinomyces had served as the sources of novel antibiotic and bioactive molecule candidates with application in many fields (Ramesh and Mathivanan, 2009; Genilloud et al., 2011). Today, the exploitation of actinomycetes with bioactive metabolites from unexplored or extreme ecosystems may be an efficient way to satisfy the everlasting demand for novel natural products, which have antimicrobial and therapeutic properties to combat human and plant pathogens (Intra et al., 2011; Ouyang et al., 2011). During the last decades, the research of actinomycetes in several alpine habitats has gained some remarkable results. For example, the study in the high Arctic permafrost of Spitsbergen has shown that there is a high diversity of actinobacterial communities in this alpine soil and many of the phylotypes identified may represent novel, uncultured species, which might be the sources of genetic diversity and ultimately novel bioactive compounds (Hansen et al., 2007). Ivanova et al. (2009) presented that a large amount of trehalose and glycerol as cryopreservation additives produced by *Streptomyces* spp. from the permafrost in Spitsbergen could be a reason for the strains to survive in cold and dry conditions. Further studies revealed that production of a range of bioactive metabolites including antibiotics, trehalose, lipase and pigment by actinobacteria from alpine cold habitats, could be a strategy for the strains in response to harsh environmental conditions (Fong et al., 2001; Dillon et al., 2003; Zhang et al., 2008; Babalola et al., 2009; Ivanova et al., 2009; Sivalingam et al., 2019). Malviya et al. (2009) documented that some *Streptomyces* isolates from alpine zones of Pindari glacier region in Indian Himalaya exhibited strong antifungal properties. These findings provide evidence that a wide diversity of Actinobacteria can survive in alpine environments, and most of which could yield bioactive compounds. In addition, as pivotal participants in the biogeochemical cycles, actinobacteria were known to possess diverse physiology and metabolic flexibility to survive in unfavorable environments (Shivlata and Satyanarayana, 2015). Thus exploring the phylogenetic and physiological diversity patterns of alpine actinomycetes may provide an opportunity for selecting strains that are under environmental pressures, which may drive adaptions that produce unique biosynthetic or hydrolytic capabilities.

The Qinghai-Tibetan Plateau, as the largest and highest plateau on Earth, possesses unique climate characteristics: alpine hypoxia, less precipitation, low humidity, osmotic stress and high incident radiation, which involves the plants and soil on the plateau in extreme harsh environmental conditions such as low temperature, anoxygenous, prolonged UV radiation and oligotrophic (Zhang et al., 2007a). Accordingly, the actinomycetes living in it may represent novel species and develop unique physiological adaption mechanism, ultimately yield bioactive compounds. However, the cultivable soil actinomycetes in this unique habitat remain relatively unexplored except for the preliminary survey on the *Potentilla fruticosa* L. alpine meadow and reports of novel actinomycetes (Wang et al., 2004; Chen et al., 2011; Zhang et al., 2016a, 2019). Thus, we had isolated and characterized actinomycetes from near-root soils of different plant communities, which distributed in two geographically diverse alpine habitats along an altitudinal gradient (2200 – 4200 m at the Laohu Ditch site; 5018 – 5130 m at the Gaize County area) on the Qinghai-Tibetan Plateau, aiming to explore the phylogenetic and physiological diversity of the cultivable actinomycetes.

## Materials and Methods

### Site Description and Sample Collection

The Laohu Ditch, located in the northeast of the Qinghai-Tibetan Plateau, is characterized by distinct vertical distribution of plant communities, from desert and desertification grassland (2200 m), mountain grassland (2800 m), alpine bushwood (3350 m), alpine meadow (3800 m), to alpine cold-desert (4200 m). The mean annual precipitation (MAP) ranged from 73.3 mm (2200 m) to 279.4 mm (4200 m), and the mean annual temperature (MAT) was from 8.1°C (2200 m) to – 5.3°C (4200 m) (offered by Qilian Mountains Station, State Key Laboratory of Cryospheric Sciences). The area of Gaize County underlain by permafrost in the central Tibetan Plateau, is distinguished by its high elevation (>5000 m) and unique plant communities adapting to this low temperature, anoxia and strong UV habitats. The MAT in the area of Gaize sampling sites was approximately 0°C with monthly mean temperature - 12.1°C in January and 12.8°C in July, and the MAP was approximately 150 mm year^–1^ (Qiao et al., 2015). The detailed information was depicted in Table 1.

**TABLE 1:** Detailed description of the sampling sites on the Qinghai-Tibetan Plateau.

Laohu Ditch				Gaize County			
Site/GPS	Altitude (m)	Dominant plant species	Vegetatio*n* type and cover	Site/GPS	Altitude (m)	Dominant plant species	Vegetatio*n* type and cover
96°11.39′; 39°44.71′	2200	*Salsola collina* Pall	DDG, 8%	85°37.694′; 33°23.506′	5130	*Dh*	Desert steppe, 10%
		*Cl*					
		*Stipa glareosa* PA Smirn					
96°24.98′; 39°37.95′	2800	*Al*	Mountain grassland, 15%	85°37.712′; 33°23.251′	5056	*Carex moocroftii* Falc. ex Boott	Alpine meadow, 80%
		*Potentilla saudersiana* Royle					
96°26.10′; 39°35.40′	3350	*Saussurea japonica* Kuntze	Alpine bushwood, 36%	85°37.579′; 33°23.156′	5100	*Kobresia pygmaea* C.B. Clarke	Alpine meadow, 80%
		*Potentilla fruticosa* L.					
		*Ll*					
		*Rq*					
96°30.28′; 39°32.05′	3800	*Au*	Alpine meadow, 41%	85°37.772′; 33°23.264′	5018	*Stipa capillata* L.	Alpine steppe, 50%
		*Arenaria kansuensis* Maxim					
		*Draba nemorosa* L.					
		*Poa annua* L.					
96°31.18′; 39°29.96′	4200	*Al*	Alpine cold-desert, 5%	85°07.650′; 33°48.080′	5020	*Stipa purpurea* Griseb.	Alpine steppe, 40%
		*Cd*					

*DDG, desert and desertification grassland; *Cl*, *Ceratoides latens* reveal & N. H. Holmgren; *Al*, *Astragalus licentianus* Hand.-Mazz.; *Ll*, *Leontopodium leontopodoides* beauverd; *Rq*, *Rhodiola quadrifida* Fisch. & C.A. Mey; *Au*, *Androsace umbellata* (Lour.) Merr.; *Cd*, *Cancrinia discoidea* Poljakov ex Tzvelev; *Dh*, *Dracocephalum heterophyllum* Benth.*

Near-root soils were sampled from fifteen dominant plants distributing along the altitude gradients in the Laohu Ditch and five distinct dominant plants in the Gaize County area in July. The samples were aseptically processed according to the previous described (Xu et al., 1996; Zhang et al., 2007a,b; Mingma et al., 2014). Briefly, five soil cores, which were 4.5 cm in diameter and 10 cm deep, were collected radially up to ∼10 cm from the base of the plant with ∼20 cm distances, mixed to form one composite soil sample. All the samples were immediately placed in sterilized ice coolers and transported to the laboratory within 24 h.

### Isolation of Actinomycetes

Actinomycetes were isolated by spreading dilutions of soil samples on petri dishes using Gao’s No. 1 medium (20 g of soluble starch, 1 g of KNO_3_, 0.5 g of K_2_HPO_4_, 0.5 g of MgSO_4_⋅7H_2_O, 0.5 g of NaCl, 0.01 g of FeSO_4_⋅7H_2_O, 20 g of agar, pH 7.2 – 7.4). The media also contained 25 μg/mL potassium dichromate to minimize bacterial and fungal contamination (Xu et al., 1996). All the plates were incubated at 20°C ± 1 for 30 days.

The growth and appearance of actinomycetes were observed every day on the medium plates and the colonies were recognized by their characteristics such as leathery or powdery appearance with concave, convex, crumpled or flate surface etc. Representative isolates of 128 that formed colonies with visually different morphologies were selected from 200 initially recovered colonies and subcultured to obtain pure colonies for further studies.

### DNA Extraction, 16S rDNA Amplification, and Sequencing

Total DNA were extracted from subcultures as described by Orsini and Romano-Spica (2001) with minor adjustment. Briefly, fresh biomass (around 50 mg) was suspended in 1 mL washing solution [50 mM Tris–HCl, pH 7.7, 25 mM EDTA, 0.1% sodium dodecyl sulfate (SDS), 0.1% polyvinylpyrrolidone (PVP)]. After centrifuging at 12,000 × *g* for 2 min, the biomass was resuspended in 100 μl lysis solution (50 mM Tris–HCl, pH 8.0, 25 mM EDTA, 3% SDS, 1.2% PVP) and heated in a microwave oven at 700 W for 45 s, then added 400 μl preheated (65°C) extraction solution (10 mM Tris–HCl, pH 8.0, 1 mM EDTA, 0.3 M sodium acetate, 1.2% PVP). The DNA pellet was phenol−chloroform extracted, precipitated in isopropyl alcohol, washed with 70% ethanol, air-dried at room temperature then resuspended in deionized distilled water for use. The universal bacterial primers 27F (5′-AGAGTTTGATCCTGGCTCAG-3′) and 1504R (5′-TTAAGGATGGTGATGCCGCA-3′) were used for amplification of 16S rDNA sequences, and the PCR amplication was performed as follows: 5 min at 95°C, followed by 30 cycles of 1 min at 95°C, 1 min at 56°C for annealing and 2 min at 72°C for extension, and a final extension for 8 min at 72°C. The PCR products were confirmed by electrophoresis in a 1% (w/v) agarose gel, stained with ethidium bromide in TAE buffer, then sent to purify and cycle sequencing using an ABI3100 automated sequencer at Beijing Sangon Biotech (Beijing, China).

### Nucleotide Sequence Accession Numbers

The 16S rDNA sequences of 128 isolates reported in this study have been submitted to the GenBank nucleotide sequence databases under accession nos. JQ812058-JQ812111 and JQ838073-JQ838150 available at https://www.ncbi.nlm.nih.gov/nucleotide.

### Phylogenetic Analysis

For further phylogenetic analysis, the sequenced 16S rDNA of the 128 isolates were matched with those in a public database using the EzBioCloud tool, and the nearest representative gene sequences of related type strains were downloaded then aligned with the isolated sequences using Clustal W program. Phylogenetic trees of the isolates were constructed by using the Maximum Likelihood method and Tamura-Nei model (Tamura and Nei, 1993) with bootstrap analysis of 1,000 replicates (Felsenstein, 1985) performed in the MEGA X package, and then the trees were edited by Evolview^1^.

### Screening of Soil Actinomycetes for Organic Acid, H_2_S and Extracellular Enzymes Production

All the 128 soil actinomycetes were examined qualitatively for the production of organic acid, H_2_S and extracellular enzymes including lipase 1, lipase 2, and lipase 3, amylase, protease, urease, catalase. Each isolate was inoculated on the center of the respective substrates such as tween 20 (lipase 1), tween 40 (lipase 2), tween 80 (lipase 3), starch (amylase), gelatin (protease) amended agar plates separately and incubated for 7–14 days at room temperature, the plates directly detected by clearing zones around the colonies were regarded as positive for enzyme activity (Sanchez-Porro et al., 2003; Ramesh and Mathivanan, 2009). The assays of urease, catalase, organic acid and H_2_S production were performed as described by Shirling and Gottlieb (1966). In addition, the diffusible pigments were also documented. Each test was conducted in triplicate, and plates or tubes with the same medium but without actinomycete isolates were maintained for controls.

### Screening of Soil Actinomycetes for Antimicrobial Activity

All the 128 soil actinomycete isolates were screened for antimicrobial activity by agar overlay method as described by Anand et al. (2006) with minor adjustment. Spore suspensions of actinomycetes were inoculated on Gao’s No. 1 medium and cultured at 20°C ± 1 for 7 days, then overlaid with 5 ml of soft nutrient agar (0.6% agar) containing 500 μL of overnight growing culture of the tested microorganisms, including *Escherichia coli* ATCC25922 representing Gram-negative bacteria, *Staphylococcus aureus* ATCC25923 representing Gram-positive bacteria, *Candida albicans* ATCC66415 representing yeast-like fungi and a clinical isolated *Pseudomonas aeruginosa* strain representing freshly pathogenic multi-resistant bacterial strain. The overlaid plates were then incubated at 28°C for 24 h and the clear inhibition zone around each isolate was recorded as positive for antimicrobial activity (Ramesh and Mathivanan, 2009). Plates with the same medium simultaneously inoculated with the tested human pathogens but without actinomycete isolates were maintained for controls.

## Results

### 16S rDNA-Based Phylogenetic Diversity of the Cultivable Actinomycetes

**FIGURE 1:**
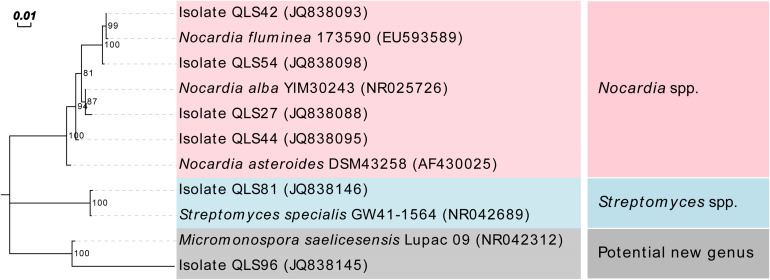
Maximum Likelihood tree based on nearly complete 16S rDNA sequences showing relationship between the isolates from the Laohu Ditch and the genera *Streptomyces* spp., *Nocardia* spp., and *Micromonospora* spp.

Seventy eight actinomycetes with representative phenotypes were recovered from the Laohu Ditch, and the 16S rDNA sequencing revealed that the predominant genus was *Streptomyces* (73 isolates), followed by *Nocardia* (4 isolates), and one isolate with only 92% sequence similarity to its nearest match *Micromonospora saelicesensis* from the GenBank database, indicating that it may be a potential novel isolate at genus level. The phylogenetic tree demonstrating the relationship between the genera *Streptomyces* spp. (randomly selected representative sequences of *Streptomyces* spp.), *Nocardia* spp., and *Micromonospora* spp. was presented on Figure 1.

**FIGURE 2:**
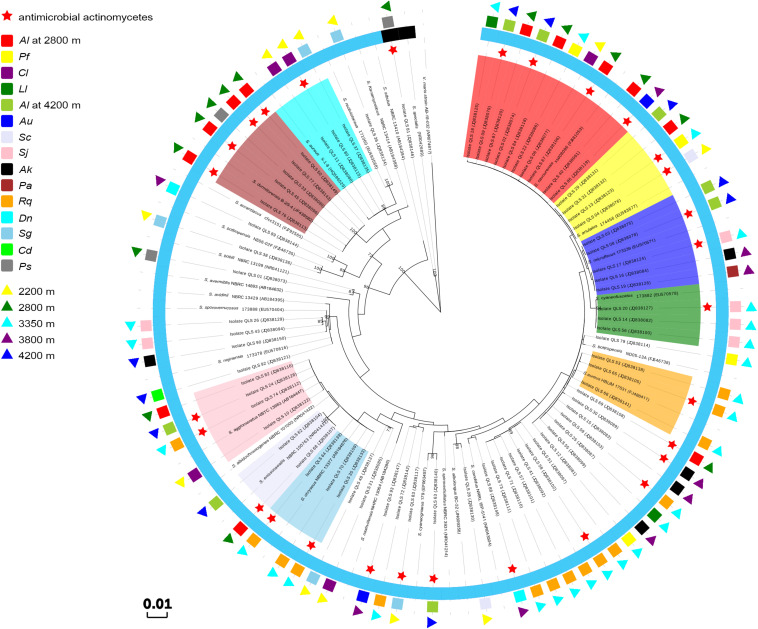
Phylogenetic tree of the *Streptomyces* isolates from the Laohu Ditch and their nearest relatives based on 16S rDNA sequences. The inner ring indicates the relationship of the *Streptomyces* spp., Cluster 1 in shade of red, Cluster 2 in shade of yellow, Cluster 3 in shade of blue, Cluster 4 in shade of green, Cluster 5 in shade of orange, Cluster 6 in shade of lavender, Cluster 7 in shade of skyblue, Cluster 8 in shade of pink, Cluster 9 in shade of brown, Cluster 10 in shade of cyan; the black semi-ring and the light blue semi-ring represent Group I and GroupII, respectively; the middle ring indicates the distribution of the 73 isolates in near-root soils of 15 plant communities. *Al*, *Astragalus licentianus* Hand.-Mazz.; *Ll*, *Leontopodium leontopodoides* Beauverd; *Pf*, *Potentilla fruticosa* L.; *Cl*, *Ceratoides latens* Reveal & N.H. Holmgren; *Au*, *Androsace umbellata* (Lour.) Merr.; *Sc*, *Salsola collina* Pall; *Sj*, *Saussurea japonica* Kuntze; *Ak*, *Arenaria kansuensis* Maxim; *Pa*, *Poa annua* L.; *Rq*, *Rhodiola quadrifida* Fisch. & C.A. Mey; *Dn*, *Draba nemorosa* L.; *Sg*, *Stipa glareosa* PA Smirn; *Cd*, *Cancrinia discoidea* Poljakov ex Tzvelev; *Ps*, *P. saudersiana* Royle; the outer ring indicates the distribution of the 73 isolates in diverse altitudes.

As the dominant isolates, a phylogenetic tree displaying the relationships between 73 *Streptomyces* isolates in this study, and between them and their nearest relatives from GenBank, was also constructed as shown in Figure 2. This allowed the sorting of the sequences into two disparate groups, designed as Groups I and II. In Group I, the single isolate QLS81 from near-root soil of *Potentilla saudersiana* Royle at 2800 m, a putatively new species of *Streptomyces* spp. to be furtherly confirmed by DNA-DNA pairing value, formed a distinct branch with the highest 16S rDNA sequence similarity of 99% to the type strain of *Streptomyces specialis*. Strikingly, it can produce substantial diffusible black pigment which biological significances needed to be furtherly explored (Supplementary Figure S1). In Group II, 52 out of the 72 *Streptomyces* isolates formed relatively distinct phyletic lines with their 16S rDNA sequences showing 99 to 100% similarities to their type strains obtained from the GenBank database. Interestingly, 42 isolates amongst the 52 isolates were sorted into ten clusters, with most of the isolates originating from the same plant assigned to the same cluster. Cluster 1 consisted of 10 isolates showing 99–100% similarity to the sequence of *Streptomyces cavourensis*. One isolate was obtained from *Ceratoides latens* Reveal & N.H. Holmgren at 2200 m, four (QLS40, QLS67, QLS84, and QLS87) from *Astragalus licentianus* Hand.-Mazz. at 2800 m, the other two (QLS02 and QLS09) from *A. licentianus* Hand.-Mazz. at 4200 m, one (QLS85) from *Androsace umbellata* (Lour.) Merr. at 3800 m, and two (QLS18 and QLS22) from *Leontopodium leontopodoides* Beauverd and *Potentilla fruticosa* L. at 3350 m, respectively. Four isolates in Cluster 2 showing 99–100% identity to *Streptomyces anulatus*, were found in *Salsola collina* Pall at 2200 m (QLS04), *A. licentianus* Hand.-Mazz. at 2800 m (QLS31), *P. fruticosa* L. at 3350 m (QLS13) and *A. licentianus* Hand.-Mazz. at 4200 m (QLS29). Cluster 3 consisting of 5 isolates displaying 99–100% identity to *Streptomyces microflavus* could be divided into two sub-clusters. Two isolates, QLS03 and QLS08 from *A. licentianus* Hand.-Mazz. at 4200 m, with brown pigmented aerial mycelium and brown diffusible pigment, respectively, formed a distinct sub-clade; While isolates QLS16 from *Arenaria kansuensis* Maxim at 3800 m, QLS17 from *Saussurea japonica* Kuntze at 3350 m, QLS19 from *Poa annua* L. at 3800 m producing pale yellow, deep yellow and labile yellow diffusible pigments, respectively, formed another sub-clade. Cluster 4 contained 3 isolates from *Saussurea japonica* Kuntze at 3350 m showing 99–100% similarity to *Streptomyces cyaneofuscatus*. And 3 isolates in Cluster 5 showing 99% identity to *Streptomyces aureus* were isolated from *Rhodiola quadrifida* Fisch. & C.A. Mey at 3350 m. Cluster 6 containing three isolates showing 99–100% similarity to *Streptomyces chryseus* were obtained from *R. quadrifida* Fisch. & C.A. Mey at 3350 m. Two isolates in Cluster 7, QLS62, and QLS68 from *A. licentianus* Hand.-Mazz. at 4200, 2800 m, respectively, demonstrated 99% similarity to *Streptomyces enissocaesilis*. Cluster 8 consisted of 4 isolates (QLS10, QLS82, QLS74, and QLS24), obtained from *Ceratoides latens* Reveal & N.H. Holmgren at 2200 m, *A. licentianus* Hand.-Mazz. at 2800 m, *R. quadrifida* Fisch. & C.A. Mey at 3350 m and *A. licentianus* Hand.-Mazz. at 4200 m, respectively, showing 99–100% identity to *Streptomyces agglomeratus*. And Cluster 9 consisted of 5 isolates showing 99–100% identity to *Streptomyces durmitorensis*, isolated from *P. saudersiana* Royle (QLS33) and *A. licentianus* Hand.-Mazz. (QLS45, QLS50, QLS76, and QLS77) at 2800 m. Cluster 10 contained three isolates from *Ceratoides latens* Reveal & N.H. Holmgren (QLS11, QLS80) and *Stipa glareosa* PA Smirn (QLS37) at 2200 m.

**FIGURE 3:**
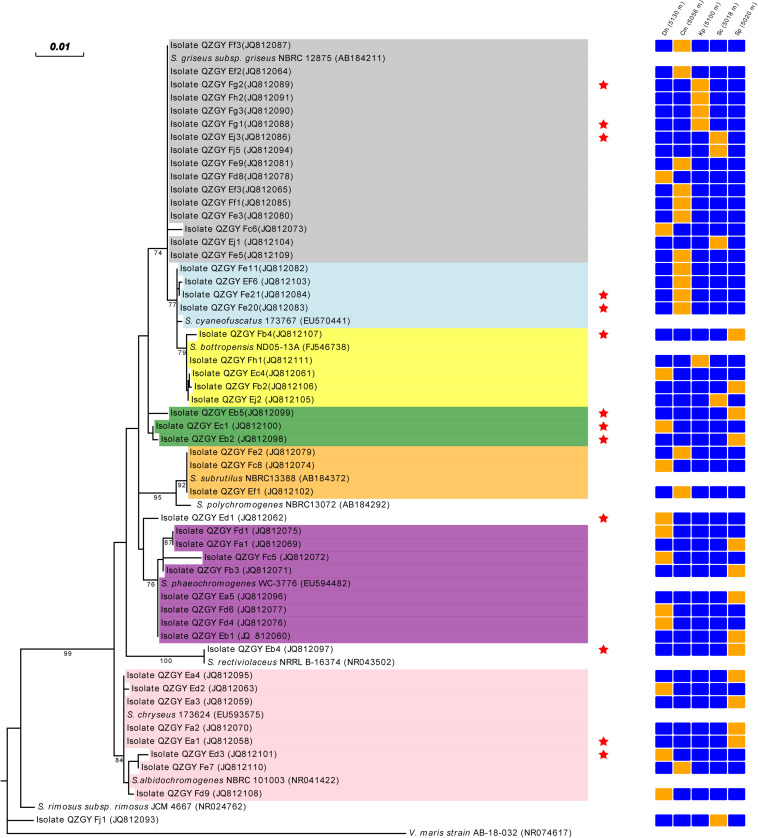
Phylogenetic tree displaying relationships of the *Streptomyces* isolates from the Gaize County area and their nearest relatives based on the 16S rDNA sequences (⋆ indicates antimicrobial actinomycetes). *Dh*, *Dracocephalum heterophyllum* Benth; *Cm*, *Carex moocroftii* Falc. ex Boott; *Kp*, *Kobresia pygmaea* C.B. Clarke; *Sp*, *Stipa purpurea* Griseb.; *Sc*, *Stipa capillata* L.; orange cell, detected; blue cell, not detected; Cluster I in shade of dark gray, Cluster II in shade of light blue; Cluster III in shade of yellow; Cluster IV in shade of green; Cluster V in shade of orange; Cluster VI in shade of purple; Cluster VII in shade of pink.

From the Gaize County area, fifty isolates with typical colony morphologies were obtained and they all pertained to *Streptomyces* spp. according to the 16S rDNA sequencing. The phylogenetic tree exhibiting the relationships between the fifty isolates, and between them and their closest relatives, was presented in Figure 3. As shown in Figure 3, isolate QZGYFj1 from *Stipa capillata* L. at 5018 m, solely formed a distinct phyletic branch, although it demonstrated 98% sequence similarity to its nearest match *Streptomyces rimosus*, indicating that it may be a potential new isolate. Isolate QZGYEb4 from *Stipa purpurea* Griseb. at 5020 m, showing 99% identity to *Streptomyces rectiviolaceus*, formed relatively phylogenetically distinct clade. While isolate QZGYEd3 solely formed relatively distinct phyletic line. The 47 remaining isolates were assigned to seven clusters. Cluster I contained 16 isolates distributing in *S. capillata* L. at 5018 m, *Carex moocroftii* Falc. ex Boott at 5056 m, *Kobresia pygmaea* C.B. Clarke at 5100 m and *Dracocephalum heterophyllum* Benth at 5130 m, exhibiting 99–100% similarity to *Streptomyces griseus*. Cluster II containing four isolates at 5056 m demonstrated 99–100% identity to *S. cyaneofuscatus*. Cluster III consisted of 5 isolates distributing at 5020, 5018, 5100, and 5130 m, showing 99–100% identity to *Streptomyces bottropensis*. Cluster IV consisted of three isolates QZGYEb5, QZGYEb2 and QZGYEc1, with isolates QZGYEc1 at 5130 m and QZGYEb2 at 5018 m forming a subclade. And three isolates in Cluster V displayed 99% similarity to *Streptomyces subrutilus*, with QZGYFe2 and QZGYEf1 at 5056 m and QZGYFc8 at 5130 m. Cluster VI contained eight isolates, with four isolates, QZGYFd1 and QZGYFc5 at 5130 m, QZGYFa1 and QZGYFb3 at 5020 m forming a sub-cluster, while another four isolates clustering together showing 99–100% similarity to *Streptomyces phaeochromogenes*. And eight isolates in Cluster VII distributed at 5020, 5056, and 5130 m, with 3 isolates forming a sub-cluster and the other 5 clustering together demonstrating 99–100% identity to *S. chryseus*.

**FIGURE 4:**
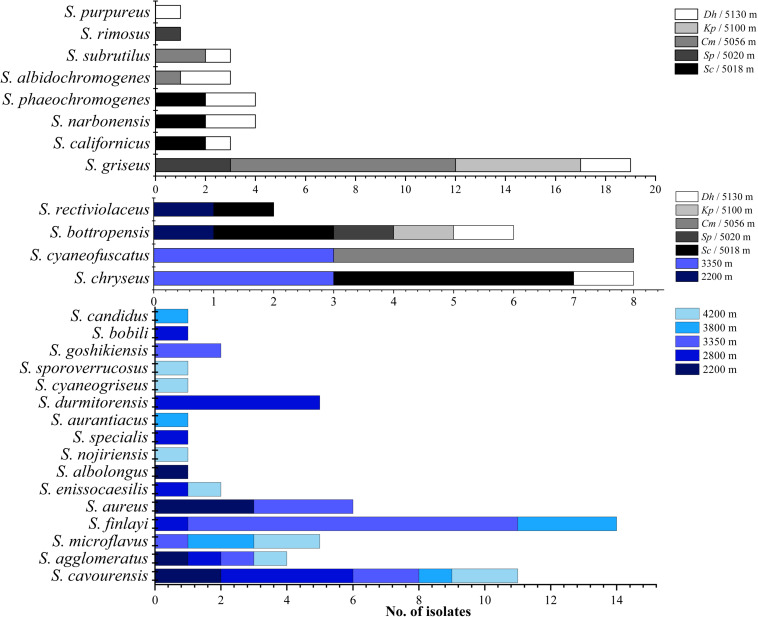
Distribution of isolates related to different *Streptomyces* species on diverse altitudes of the Laohu Ditch and Gaize County area.

As shown in Figures 2–4, isolates with 99–100% 16S rDNA sequence similarity to *S. cavourensis* widely distributed in different plant communities across five altitudes on the Laohu Ditch. While isolates with 99–100% 16S rDNA sequence identity to *S. griseus* predominated among the isolates on the Gaize County area, constituting 32% of the total isolates. In addition, isolates with 99–100% 16S rDNA sequence identities to *S. rectiviolaceus*, *S. chryseus*, *S. cyaneofuscatus* and *S. bottropensis* occurred both in the Laohu Ditch and Gaize County area. However, variances of *Streptomyces* isolates in the Laohu Ditch and Gaize County area were observed. For example, isolates with 99–100% 16S rDNA sequence identities to *S. specialis*, *Streptomyces goshikiensis*, *Streptomyces aurantiacus*, *Streptomyces nojiriensis*, *Streptomyces purpureus* only occurred at 2800, 3350, 3800, 4200, and 5130 m, respectively.

### Phenotypic Characteristics of the Cultivable Actinomycetes

**FIGURE 5:**
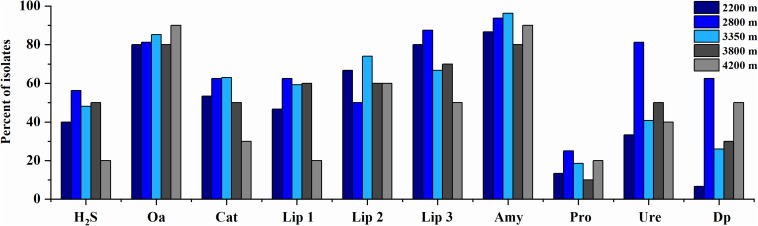
Distribution of H_2_S, organic acid, diffusible pigments and various enzymes producing isolates at the increasing altitudes of the Laohu Ditch. Cat, catalase; Lip 2, lipase 2; Ure, urease; Pro., protease; Lip 3, lipase 3; Amy, amylase; Lip 1, lipase 1; Dp, diffusible pigment; Oa, organic acid.

**FIGURE 6:**
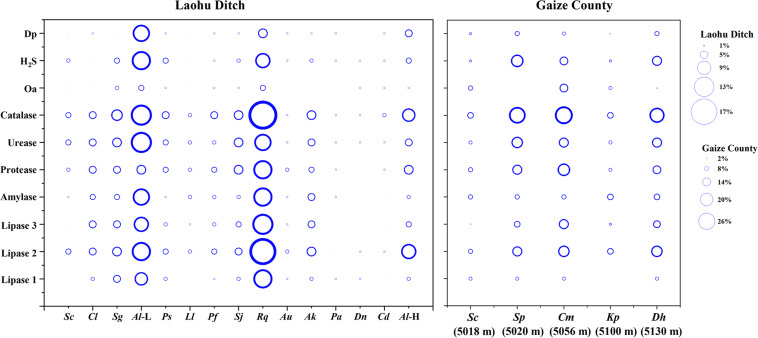
Distribution of H_2_S, organic acid, diffusible pigment and various enzymes producing isolates in near-root soils of twenty plant communities on the Tibetan Plateau. (The size of the bubble indicates the proportion of organic acid, H_2_S, diffusible pigment and various enzymes producing isolates to the total isolates, respectively). *Al* (L), *Astragalus licentianus* Hand.-Mazz. at 2800 m, *Al* (H), *Astragalus licentianus* Hand.-Mazz. at 4200.

The phenotypic properties of the isolates were shown in Figures 5, 6. From the Laohu Ditch, more than half of the 78 isolates produced catalase, lipase 2, urease, gelatinase, lipase 3 amylase. H_2_S, lipase 1, pigment and organic acid producing isolates accounted for 48.7, 44.9, 33.3, and 17.9% of the total isolates, respectively. As shown in Figure 5, H_2_S, organic acid, diffusible pigment and various extracellular enzymes producing isolates were widely distributed in the five altitudes, with diffusible pigment, urease, H_2_S, lipase 1, lipase 3 and protease producing isolates dominating at 2800 m, amylase and lipase 1 producing isolates predominant at 3350 m, organic acid producing isolates dominating at 4200 m. However, as displayed in Figure 6 the proportion of H_2_S, organic acid, diffusible pigment and extracellular enzymes producing isolates demonstrated great discrepancy in near-root soils of different plant communities. Catalase and urease producing isolates were distributed across 15 plant communities; lipase 2, protease producing isolates occurred in 14 plants; amylase, H_2_S, diffusible pigment producing isolates occurred in 13 plants, and lipase 1, lipase 3 in 12 plants, organic acid producing isolates only occurred in 10 plants. In addition, H_2_S, organic acid, diffusible pigment and various enzymes producing isolates frequently occurred in two medicinal plants, *A. licentianus* Hand.-Mazz. and *R. quadrifida* Fisch. & C.A. Mey.

Among 50 *Streptomyces* isolates from the Gaize County area, more than half of the isolates produced catalase, lipase 2, urease, protease, H_2_S. Amylase, lipase 3, lipase 1, pigment and organic acid producing isolates accounted for 46, 46, 24, 28, and 30% of the total isolates, respectively. As shown in Figure 6 organic acid and lipase 1 producing isolates occurred across four plant communities except for *Stipa purpurea* Griseb. at 5020 m and *Kobresia pygmaea* C.B. Clarke at 5100 m, respectively. The other eight were found in all of the five plant communities, with discrepancy in proportion and variety.

### Antimicrobial Activity of the Cultivable Actinomycetes

The antimicrobial activity varied among the actinomycetes as shown in Table 2. Among 78 actinomycete isolates from the Laohu Ditch, 29 isolates (37.2%) exhibited antimicrobial activity against the human pathogens. Of which, 2, 19, 14, 1 isolates demonstrated antimicrobial activity against *E*. *coli*, *S. aureus*, *C*. *albicans*, and *P*. *aeruginosa*, respectively. In addition, 7 isolates showed antimicrobial activity against two of the four human pathogens. And the 29 antagonistic actinomycetes were all screened from *Streptomyces* spp. As shown in Figure 2, they were widely spread in distinct phylogenetic clusters from 12 plant communities at different altitudes. Noticeably, 51.4% of the tested isolates (35 in total) from near-root soils of two medicinal plants (*A. licentianus* Hand.-Mazz. and *R. quadrifida* Fisch. & C.A. Mey) demonstrated antimicrobial activity, which comprised 62% of the total antagonistic isolates.

**TABLE 2:** Antimicrobial activity of the actinomycetes from the alpine habitats on the Tibetan Plateau.

Laohu Ditch							Gaize County					

Altitude (m)	Plant species	Isolate no.	Antimicrobial activity				Altitude (m)/plant species	Isolate no.	Antimicrobial activity			
			*E. coli*	*S. aureus*	*C. albicans*	*P*. *aeruginosa*			*E. coli*	*S. aureus*	*C. albicans*	*P. aeruginosa*
2200	*Sc*	QLS04	**−**	**−**	**−**	+	5020/*Sp*	QZGYEa1	+	**−**	**−**	**−**
	*Cl*	QLS80	**−**	**−**	+	**−**						
	*Sg*	QLS83	**−**	**−**	+	**−**		QZGYEb2	+	+	**−**	**−**
2800	*Al*	QLS50	**−**	+	+	**−**						
	*Al*	QLS76	**−**	+	**−**	**−**		QZGYEb4	**−**	+	+	**−**
	*Al*	QLS77	**−**	+	+	**−**						
	*Al*	QLS68	**−**	+	**−**	**−**		QZGYEb5	**−**	**−**	+	**−**
	*Al*	QLS67	**−**	**−**	+	**−**						
	*Al*	QLS69	**−**	+	**−**	**−**		QZGYFb4	**−**	+	**−**	**−**
	*Ps*	QLS81	+	**−**	+	**−**						
3350	*Sj*	QLS17	**−**	**−**	+	**−**	5018/*Sc*	QZGYE j3	**−**	**−**	+	**−**
	*Sj*	QLS20	**−**	+	**−**	**−**						
	*Pf*	QLS13	**−**	+	**−**	**−**	5056/*Cm*	QZGYFe20	+	+	**−**	**−**
	*Rq*	QLS35	**−**	+	**−**	**−**						
	*Rq*	QLS65	**−**	+	**−**	**−**	5100/*Kp*	QZGYFe21	**−**	+	**−**	**−**
	*Rq*	QLS58	**−**	+	**−**	**−**						
	*Rq*	QLS59	**−**	+	**−**	**−**		QZGYFg1	**−**	+	**−**	**−**
	*Rq*	QLS64	**−**	+	**−**	**−**						
	*Rq*	QLS74	**−**	+	**−**	**−**		QZGYFg2	**−**	**−**	**−**	+
3800	*Au*	QLS85	**−**	+	+	**−**						
	*Au*	QLS93	**−**	**−**	+	**−**	5130*/Dh*	QZGYEc1	**−**	+	+	**−**
	*Ak*	QLS30	**−**	+	**−**	**−**						
	*Dn*	QLS88	**−**	+	+	**−**						
4200	*Al*	QLS02	**−**	+	+	**−**		QZGYEd1	**−**	+	+	**−**
	*Al*	QLS09	**−**	**−**	+	**−**						
	*Al*	QLS08	**−**	+	+	**−**						
	*Al*	QLS24	**−**	+	**−**	**−**		QZGYEd3	**−**	+	**−**	**−**
	*Al*	QLS45	+	**−**	**−**	**−**						
	*Al*	QLS63	**−**	**−**	+	**−**						

*+, antimicrobial activity; −, no antimicrobial activity; *Sc*, *Salsola collina* Pall; *Cl*, *Ceratoides latens* Reveal & N.H. Holmgren; *Sg*, *Stipa glareosa* PA Smirn; *Al*, *Astragalus licentianus* Hand.-Mazz.; *Ps*, *Potentilla saudersiana* Royle; *Sj*, *Saussurea japonica* Kuntze; *Pf*, *Potentilla fruticosa* L.; *Rq*, *Rhodiola quadrifida* Fisch. & C.A. Mey; *Au*, *Androsace umbellata* (Lour.) Merr.; *Ak*, *Arenaria kansuensis* Maxim; *Dn*, *Draba nemorosa* L.; *Sp*, *Stipa purpurea* Griseb.; *Sc* (5018 m): *Stipa capillata* L.; *Cm*, *Carex moocroftii* Falc. ex Boott; *Kp*, *Kobresia pygmaea* C.B. Clarke; *Dh*, *Dracocephalum heterophyllum* Benth.*

While from the Gaize County area, thirteen of the fifty actinomycete isolates (26.0%) showed growth inhibitory activity against the human pathogens. Among them, 3, 9, 5, 1 isolates displayed antimicrobial activity against *E*. *coli*, *S. aureus*, *C. albicans*, and *P. aeruginosa*, respectively. Moreover, five isolates showed a broad antimicrobial spectrum against two of the four human pathogens. As shown in Table 2, antagonistic actinomycetes were scattered around five plant communities, with the most occurring in *Stipa purpurea* Griseb. at 5020 m.

In this study, the distinct phenotypic characters and antimicrobial activities were detected among isolates with identical 16S rDNA sequences as shown in Table 3. Though apparent division of the isolates to different clusters in accordance with the 16S rDNA sequences, there was just as much physiological variation among the isolates within the same clusters obtained from the same area, as among the isolates with identical 16S rDNA sequences but in disparate area, such as isolates with 99–100% 16S rDNA sequence identities to *S. cavourensis*, *S. griseus*, and *S. rectiviolaceus* (Table 3), *S. chryseus*, *S. cyaneofuscatus*, and *S. bottropensis* (Supplementary Tables 1–3).

**TABLE 3:** Physiological heterogeneity between different isolates of the same clusters in the same area or disparate area.

Streptomycetes	*Streptomyces cavourensis*					*Streptomyces griseus*				*Streptomyces rectiviolaceus*	
			
Isolate no.	QLS06	QLS87	QLS18	QLS85	QLS02	QZGYEj3	QZGYFe9	QZGYFg1	QZGYFd8	QLS36	QZGYEb4
			
Source of isolates	*Cl* (2200 m)	*Al* (2800 m)	*Ll* (3350 m)	*Au* (3800 m)	*Al* (4200 m)	*Sc* (5018 m)	*Cm* (5056 m)	*Kp* (5100 m)	*Dh* (5130 m)	*Sg* (2200 m)	*Sp* (5020 m)
**Phenotypic character**											
Lipase 1	−	+	−	+	−	+	−	−	+	+	−
Lipase 2	+	+	+	+	+	+	−	+	+	+	+
Lipase 3	+	+	+	+	+	−	−	−	+	+	+
Amylase	+	+	+	+	+	+	+	+	+	+	+
Protease	+	−	+	+	+	+	−	−	−	+	+
Urease	−	+	+	+	+	+	−	−	−	+	+
Catalase	+	+	+	+	+	+	+	+	+	+	+
Organic acid	+	−	−	−	−	+	+	+	+	+	−
H_2_S production	+	+	−	+	−	−	+	−	+	+	+
Diffusible pigment	brown	brown	−	brown	yellow	−	−	purple	brown	−	−
**Antimicrobial activity**											
*Escherichia coli*	−	−	−	−	−	−	−	−	+	−	−
*Staphylococcus aureus*	−	−	−	+	+	−	−	+	−	−	+
*Candida albicans*	−	−	−	+	+	+	−	−	−	−	+
*Pseudomonas aeruginosa*	−	−	−	−	−	−	−	−	−	−	−

*+, positive effect; −, negative effect.*

## Discussion

Actinomycetes remain a mainstream source of antibiotics against constantly emerging multidrug resistant pathogenic microorganisms (Tiwari and Gupta, 2013; Shivlata and Satyanarayana, 2015; Barka et al., 2016). To raise the screening efficiency of valuable strains, knowledge about the diversity, physiological activity and ecological distribution of unexploited actinomycete flora is urgently needed (Tiwari and Gupta, 2013). Our study added to this field by displaying the phylogenetic diversity and physiological heterogeneity of the cultivable actinomycetes from near-root soils of different plant communities at the increasing altitudes on the Qinghai-Tibetan Plateau.

It is notable that the 16S rDNA sequence as the sole marker for phylogenetic and taxonomic analysis is limited. In fact, it has been reported that *Streptomyces* isolates with 97% 16S rDNA sequence identities can vary by as much as 30% in core genome divergence with average nucleotide identities ranging from 100–78.3% (Chevrette et al., 2019). However, the 16S rDNA sequence is widely used as an indispensable gene marker in the bacterial taxonomic analysis (Martina et al., 2019). In this study, the 128 isolates were assigned to *Streptomyce* spp., *Nocardia* spp. and one potential new genus based on the 16S rDNA sequences. Moreover, the 97% 16S rDNA sequence identity threshold has been extensively used as a boundary for bacterial species delineation (Meier-Kolthoff et al., 2013), however, this threshold value has been raised to 99% for actinomycetes (Stach et al., 2003; Guo et al., 2015) based on comparative studies between 16S rDNA sequence identities, average nucleotide identity (ANI) values of whole genomes and DNA–DNA hybridization (DDH) (Guo et al., 2015). Accordingly, we empolyed a threshold of 99–100% to assign *Streptomyces* isolates to different clusters. Surely, based on this metric, the isolates identified as potential novel isolates according to the 16S rDNA sequences deserved to be furtherly confirmed by polyphasic taxonomic approach (Zhang et al., 2019).

*Streptomyces* spp. possess high adaptive capability for surviving in many extreme environmental conditions and their incidence had been documented in diverse extreme habitats including frozen soils, deserts, oceans, Arctic and Antarctic regions (Maldonado et al., 2008; Le Roes-Hill et al., 2009; Malviya et al., 2009; Okoro et al., 2009; Ramesh and Mathivanan, 2009; Verma et al., 2009; Ivanova et al., 2010; Zothanpuia et al., 2018). Studies in frozen soils, Arctic and Antarctic regions had revealed a great diversity of cultivable *Streptomyces* species (Le Roes-Hill et al., 2009; Ivanova et al., 2010; Li et al., 2011; Zhang et al., 2016b; Kamjam et al., 2019), while in marine and deserts, *Streptomyces* spp. had been reported as one of the dominant culturable genera (Okoro et al., 2009; Prieto-Davo et al., 2016). In this study, *Streptomyces* isolates were isolated from near-root soils of the studied plant communities from an altitude of 2200 to 5130 m on the Qinghai-Tibetan Plateau, with variances in diversity on different sites. By contrast, four *Nocardia* isolates and one potential novel genus just detected in *Salsola collina* Pall of 2200 m (QLS27, QLS44), *Stipa glareosa* PA Smirn of 2200 m (QLS96), *P. saudersiana* Royle of 2800 m (QLS42) and *Arenaria kansuensis* Maxim of 3800 m (QLS54) from the Laohu Ditch. This may be attributed to high dependency of the rare actinomycetes (*Nocardia* spp. and the potential new genus) upon their living conditions, with a relatively narrow ecological niche (Horner-Devine et al., 2004). Additionally, differences in the basic nutrients such as energy, carbon and nitrogen sources necessary for actinomycetes supplied by vegetation litter and/or plant root exudates, could cause variation in actinobacterial composition (Rasche et al., 2011; Zhang et al., 2013). For example, isolates with 98–100% 16S rDNA sequence identities to 28 species of *Streptomyces* spp. were detected in 20 plant communities along different altitudes on the Tibetan Plateau. Compared to the frequent occurrence of members related to *S. cavourensis* and *S. griseus* in different plant communities at the increasing altitudes, quite a few *Streptomyces* isolates specifically occurred in near-root soils of diverse plant communities at varying altitudes. This finding implied that a high degree of *Streptomyces* spp. diversity could adapt to the extreme alpine habitats and different plants may be selective to their root-associated *Streptomyces* isolates (Adil et al., 2017; Naylor et al., 2017). Noticeably, isolates with 99–100% 16S rDNA sequence identities to *S. chryseus*, *S. rectiviolaceus*, *S. bottropensis*, and *S. cyaneofuscatus* were detected in both of the studied geographically diverse cold environments. Furthermore, based on the same taxonomy according to the 16S rDNA sequences, the occurrence of some *Streptomyces* isolates in the studied extreme alpine habitats was observed in other extreme environments as well. For example, members related to *S. griseus* and *S. rimosus* had been isolated even from thermophilic environment (Shivlata and Satyanarayana, 2015). Isolate ISP 5300 affiliated to *S. cavourensis* had been reported as an alkaliphilic *Streptomyces* species by Mikami et al. (1982). And two isolates M-169 and M-157 related to *S. cyaneofuscatus* explored in deep-sea were found to produce novel antimicrobial and antitumor compounds (Ortiz-López et al., 2018; Rodríguez et al., 2018).

In this study, almost all of the *Streptomyces* isolates from the alpine habitats produced catalase, which was consistent with the catalase-positive of *Streptomyces* spp. described in Bergey’s Manual of Systematic Bacteriology (Ruan, 2013). Colony pigment or diffusible pigment produced by bacteria is a physiological strategy of adaption to low temperature and of resistant to environmental stress (Dillon et al., 2003; Ponder et al., 2005; Zhang et al., 2008). For example, microorganisms in diverse cold habitats would increase carotenoid production to keep membrane stabilization at lower temperature and secrete dark pigment to absorb UV light (Fong et al., 2001; Dillon et al., 2003; Zhang et al., 2008). Marine actinomycetes producing diffusible pigments were able to survive longer than those with no diffusible pigment production (Ramesh and Mathivanan, 2009). In our study, forty *Streptomyces* isolates obtained from the two alpine habitats produced diffusible pigments, which may be of significance for the isolates adapting to the alpine habitats on the Tibetan Plateau. In addition, as saprophytic inhabitants, soil actinomycetes thrive on decomposing organic materials such as lignin, chitin, cellulose, sulfocompounds etc., which is enabled by means of producing diverse extracellular hydrolytic enzymes (Bhatnagar and Kim, 2010; Suela Silva et al., 2013). The result of biochemical assays for the obtained actinomycetes showed that lipase 2, urease, protease, amylase and H_2_S producing strains accounted for 82.1, 70.5, 62.8, 52.6, and 48.7% of the total isolates from the Laohu Ditch, while 72, 62, 66, 46, and 60% of the total isolates from the Gaize County area, respectively. This demonstrated that most of the *Streptomyces* isolates possessed the capability of hydrolyzing activity on polyesters, urea, gelatin, amylum and sulfur-containing amino acids, reflecting that those isolates may associate with the cyclings of carbon, nitrogen and sulfur on the Tibetan Plateau (Shivlata and Satyanarayana, 2015; Barka et al., 2016). However, variances in proportion of H_2_S, diffusible pigment, organic acid and enzymes producing isolates in each plant community at increasing altitudes on the Tibetan Plateau were detected, implying the differences in soil ingredient of diverse plant root habitats closely relate to distinct plant litter and/or root exudates (Goodfellow and Williams, 1983; Bais et al., 2006). For example, the enrichment of H_2_S, diffusible pigment, organic acid and enzymes producing isolates in near-root soils of two medicinal plants, *A. licentianus* Hand.-Mazz. and *R. quadrifida* Fisch. & C.A. Mey, was observed. Studies have shown that medicinal plant roots are rich in bioactive compounds that could affect the physiological activities of their root-associated actinomycetes (Li et al., 2008; Zhao et al., 2012; Patrycja et al., 2015; Martina et al., 2019).

Moreover, the physiological heterogeneity of some *Streptomyces* isolates was detected on the Tibetan Plateau. For example, 13.7% of the total *Streptomyces* isolates related to *S. cavourensis* frequently occurring at different plant communities across five altitudes on the Laohu Ditch, and 32% of the total isolates in the Gaize County area affiliated with *S. griseus* across four sites, exhibited distinct physiological capabilities. Most notably, with the same taxonomy according to the 16S rDNA sequences, isolate QZGYEb4 from 5020 m on the Gaize County area showed diverse antimicrobial activity and phenotypic traits from its counterpart QLS36 from 2200 m on the Laohu Ditch. The study of marine actinomycetes also found that though nearly or fully identical 16S rDNA sequences to known terrestrial organisms, the actinomycetes still have signs of adaption to their marine environment (Bredholt et al., 2008). Zhang et al. (2008) revealed that physiological variation between strains with close evolutionary relationships suggested the differences in the ecological conditions of bacteria survival habitats.

It has been demonstrated that actinomycetes in extreme environments are capable of yielding antagonistic bioactive compounds (Shivlata and Satyanarayana, 2015; Sivalingam et al., 2019). Twenty nine actinomycetes from the Laohu Ditch and thirteen from the Gaize County area, respectively, exhibiting antimicrobial activity against four kinds of pathogenic microorganisms, were all screened from *Streptomyces* spp., which were widely spread at the increasing altitudes of the studied areas on the Tibetan Plateau. Interestingly, the antagonistic isolates still slightly enriched in the near-root soils of two medicinal plants, *A. licentianus* Hand.-Mazz. (28.6% of the total antagonistic streptomycetes) and *R. quadrifida* Fisch. & C.A. Mey (14.3% of the total antagonistic streptomycetes). Barakate et al. (2002) presented that 14 and 68% of the 131 streptomycetes from plant rhizospheric soils showed antimicrobial activity against *Escherichia coli* and *Staphylococcus aureus*, respectively. Several researchers had reported antimicrobial activity of actinomycetes against various pathogenic microorganisms from other unique habitats. Thirty nine actinomycetes recovered from the Antarctic soils, of which, fifteen displayed antagonistic activity against clinical Gram-positive and Gram-negative bacteria (Lee et al., 2012). Ramesh and Mathivanan (2009) obtained 208 marine actinomycetes, of which, 14.9, 8.7, and 13.5% exhibited antimicrobial activity against *E*. *coli*, *P. aeruginosa* and *C. albicans*, respectively. These results revealed that the unexplored unique habitats have the potential to discover actinomycetes with antimicrobial activities as well as various bioactive compounds.

To the best of our knowledge, this study offers for the first time a prelude about the unexplored culturable soil actinomycetes diversity associated with the typical alpine habitats on the Qinghai-Tibetan Plateau and their bioactive capabilities. However, more in-depth investigations on extraction, purification of the bioactive compounds produced by the actinomycetes, as well as the cold adaption mechanism of actinomycetes in alpine habitats will strengthen the development and utilization of those actinomycete isolates.

## Data Availability Statement

The datasets presented in this study can be found in online repositories. The names of the repository/repositories and accession number(s) can be found in the article/Supplementary Material.

## Author Contributions

SX, XZ, and CZ planned and designed the research. KJ, JLL, MDW, and YW provided the help in sampling. MMW and JHL analyzed the data. AM conducted the experiments and wrote the manuscript. All authors were involved in revising the manuscript critically.

## Conflict of Interest

The authors declare that the research was conducted in the absence of any commercial or financial relationships that could be construed as a potential conflict of interest.

## Footnotes

1 http://www.evolgenius.info/evolview
