# Supplementary material for: Phylogenetic and Physiological Diversity of Cultivable Actinomycetes Isolated From Alpine Habitats on the Qinghai-Tibetan Plateau
Source: Front Microbiol. 2020 Oct 2;11:555351. doi: 10.3389/fmicb.2020.555351 (PMC7566193; doi:10.3389/fmicb.2020.555351)
Supplement: Supplementary file 2 [file Data_Sheet_1.docx]

**Phylogenetic and physiological diversity of cultivable actinomycetes isolated from alpine habitats on the Qinghai-Tibetan plateau**

Aiai Ma ^1, 2^, Xinfang Zhang ^1^, Kan Jiang ^3^, Changming Zhao ^1^**,** Junlin Liu ^2^, Mengdan Wu ^3^, Ying Wang ^4^, Mingming Wang ^2^, Jinhui Li ^1^, Shijian Xu ^1, *^

1. School of Life Sciences, Lanzhou University, Lanzhou, Gansu 730000, China.
2. Life Science and Engineering College of Northwest University for Nationalities, Lanzhou, Gansu 730000, China.
3. College of Agronomy, Gansu Agricultural University, Lanzhou, 730070, China.

*Corresponding author: Tel: 13919170189.

*E-mail address*: xushijian@lzu.edu.cn

**Supplementary** Table 1 Physiological diversity between different isolates related to *Streptomyces chryseus* from disparate area

| Streptomycetes | |  | *Streptomyces chryseus* | | | | | | | | |
| --- | --- | --- | --- | --- | --- | --- | --- | --- | --- | --- | --- |
| Source of isolates | |  | Laohu Ditch | | |  | Gaize County | |  |  |  |
| Isolate no. | |  | QLS35 | QLS64 | QLS70 |  | QZGYEa1 | QZGYEa3 | QZGYEa4 | QZGYFa2 | QZGYEd2 |
| **Phenotypic character** | |  |  |  |  |  |  |  |  |  |  |
| Lipase 1 |  |  | **－** | **－** | + |  | **－** | **－** | + | **－** | **－** |
| Lipase 2 |  |  | + | + | + |  | + | **－** | + | + | + |
| Lipase 3 |  |  | + | **－** | **－** |  | + | **－** | + | **－** | + |
| Amylase |  |  | **－** | + | **－** |  | **－** | **－** | **－** | **－** | **－** |
| Protease |  |  | **－** | **－** | + |  | + | + | **－** | **－** | + |
| Urease |  |  | **－** | + | + |  | + | **－** | + | + | + |
| Catalase |  |  | + | + | + |  | + | + | + | + | + |
| Organic acid |  |  | **－** | **－** | **－** |  | **－** | **－** | **－** | **－** | **－** |
| H_2_S production | |  | + | + | + |  | + | + | + | + | + |
| Pigment |  |  | yellow | yellow | **－** |  | yellow | **－** | yellow (labile) | **－** | yellow |
| **Antimicrobial activity** | |  |  |  |  |  |  |  |  |  |  |
| *Escherichia coli* | |  | **－** | **－** | **－** |  | + | **－** | **－** | **－** | **－** |
| *Staphylococcus aureus* | |  | + | + | **－** |  | **－** | **－** | **－** | **－** | **－** |
| *Candida albicans* | |  | **－** | **－** | **－** |  | **－** | **－** | **－** | **－** | **－** |
| *Pseudomonas aeruginosa* | | | **－** | **－** | **－** |  | **－** | **－** | **－** | **－** | **－** |

+: positive effect; －: negative effect.

**Supplementary** Table 2 Physiological diversity among different isolates related to *Streptomyces cyaneofuscatus* from disparate area

| Streptomycetes | |  | *Streptomyces cyaneofuscatus* | | | | | | | |
| --- | --- | --- | --- | --- | --- | --- | --- | --- | --- | --- |
| Source of isolates | |  | Laohu Ditch | | |  | Gaize County | |  |  |
| Isolate no. | |  | QLS14 | QLS20 | QLS56 |  | QZGYEf6 | QZGYFe11 | QZGYFe20 | QZGYFe21 |
| **Phenotypic character** | |  |  |  |  |  |  |  |  |  |
| Lipase 1 |  |  | + | **－** | **－** |  | **－** | + | + | **－** |
| Lipase 2 |  |  | + | **－** | + |  | + | + | + | + |
| Lipase 3 |  |  | + | **－** | + |  | + | + | **－** | + |
| Amylase |  |  | **－** | **－** | + |  | **－** | **－** | **－** | **－** |
| Protease |  |  | + | + | + |  | + | + | + | + |
| Urease |  |  | + | + | + |  | + | + | + | + |
| Catalase |  |  | + | **－** | + |  | + | + | + | + |
| Organic acid |  |  | **－** | **－** | **－** |  | + | **－** | + | + |
| H_2_S production | |  | **－** | **－** | + |  | + | **－** | + | + |
| Diffusible pigment |  |  | **－** | **－** | **－** |  | **－** | **－** | **－** | brown |
| **Antimicrobial activity** | |  |  |  |  |  |  |  |  |  |
| *Escherichia coli* | |  | **－** | **－** | **－** |  | **－** | **－** | + | **－** |
| *Staphylococcus aureus* | |  | **－** | + | **－** |  | **－** | **－** | + | + |
| *Candida albicans* | |  | **－** | **－** | **－** |  | **－** | **－** | **－** | **－** |
| *Pseudomonas aeruginosa* | | | **－** | **－** | **－** |  | **－** | **－** | **－** | **－** |

+: positive effect; －: negative effect.

**Supplementary** Table 3 Physiological diversity among different isolates related to *Streptomyces bottropensis* from disparate area

| Streptomycetes | |  | *Streptomyces bottropensis* | | | | | | |
| --- | --- | --- | --- | --- | --- | --- | --- | --- | --- |
| Source of isolates | |  | LaohuDitch |  | Gaize County | |  |  |  |
| Isolate no. | |  | QLS79 |  | QZGYEc4 | QZGYEj2 | QZGYFb2 | QZGYFb4 | QZGYFh1 |
| **Phenotypic character** | |  |  |  |  |  |  |  |  |
| Lipase 1 |  |  | **－** |  | **－** | + | **－** | **－** | **－** |
| Lipase 2 |  |  | + |  | + | + | + | + | + |
| Lipase 3 |  |  | + |  | + | **－** | + | **－** | + |
| Amylase |  |  | **－** |  | **－** | + | **－** | **－** | + |
| Protease |  |  | + |  | + | + | + | + | + |
| Urease |  |  | **－** |  | + | **－** | + | **－** | + |
| Catalase |  |  | + |  | + | + | + | + | + |
| Organic acid |  |  | + |  | **－** | + | **－** | **－** | **－** |
| H_2_S production | |  | + |  | + | **－** | + | + | + |
| Diffusible pigment |  |  | brown |  | **－** | **－** | **－** | **－** | **－** |
| **Antimicrobial activity** | |  |  |  |  |  |  |  |  |
| *Escherichia coli* | |  | **－** |  | **－** | **－** | **－** | **－** | **－** |
| *Staphylococcus aureus* | |  | **－** |  | **－** | **－** | **－** | + | **－** |
| *Candida albicans* | |  | **－** |  | **－** | **－** | **－** | **－** | **－** |
| *Pseudomonas aeruginosa* | | | **－** |  | **－** | **－** | **－** | **－** | **－** |

+: positive effect; －: negative effect.
